# The Ethics of Electronic Tracking Devices in Dementia Care: An Interview Study with Developers

**DOI:** 10.1007/s11948-024-00478-0

**Published:** 2024-05-08

**Authors:** Jared Howes, Yvonne Denier, Tijs Vandemeulebroucke, Chris Gastmans

**Affiliations:** 1 Centre for Biomedical Ethics and Law, KU Leuven, Leuven, Belgium; 2 Sustainable AI Lab, Institute for Science and Ethics, University of Bonn, Bonn, Germany

**Keywords:** Dementia, Wandering, Ethics, Development, Location-based technologies

## Abstract

Wandering is a symptom of dementia that can have devastating consequences on the lives of persons living with dementia and their families and caregivers. Increasingly, caregivers are turning towards electronic tracking devices to help manage wandering. Ethical questions have been raised regarding these location-based technologies and although qualitative research has been conducted to gain better insight into various stakeholders' views on the topic, developers of these technologies have been largely excluded. No qualitative research has focused on developers’ perceptions of ethics related to electronic tracking devices. To address this, we performed a qualitative semi-structured interview study based on grounded theory. We interviewed 15 developers of electronic tracking devices to better understand how they perceive ethical issues surrounding the design, development, and use of these devices within dementia care. Our results reveal that developers are strongly motivated by moral considerations and believe that including stakeholders throughout the development process is critical for success. Developers felt a strong sense of moral obligation towards topics within their control and a weaker sense of moral obligation towards topics outside their control. This leads to a perceived moral boundary between development and use, where some moral responsibility is shifted to end-users.

**Supplementary Information:**

The online version contains supplementary material available at 10.1007/s11948-024-00478-0.

## Introduction

Dementia is a term for a variety of neuro-degenerative diseases or conditions that disrupt and impair daily life and independence (Robinson et al., 2015). As the stages of dementia progress, persons with dementia (PwD) will increasingly rely on others for their needs, and typically will require institutionalized care at some point (Toot et al., 2017). Wandering is a behavioral symptom of dementia characterized by frequent, repetitive, temporally-disordered and/or spatially-disoriented movements that are often expressed via lapping, random, or pacing movements (Algase et al., 2007). Wandering behavior may lead to elopement, elopement attempts, or getting lost (Moore et al., 2009). Not all wandering is dangerous, but PwDs who elope or become lost have a higher risk for serious injury or death (Lissemore et al., 2019; Bantry White & Montgomery, 2015). Additionally, incidences of wandering can spur institutionalization of PwDs, even if they prefer to remain living at home (Rapaport et al., 2020).

Caregivers are increasingly turning towards technology to help manage and prevent wandering related harms (Neubauer et al., 2018). Electronic tracking devices, also known as locators, monitors, personal safety alarms, or surveillance devices, are devices that facilitate the monitoring, locating, or logging of a person with dementia’s temporal location. They can come packaged with a combination of technologies in different form factors, such as a GPS watch, or an RIFD bracelet, amongst others. These devices have great potential in helping monitor PwDs, enabling caregivers to intervene and quickly locate a lost individual. That these devices touch upon ethically sensitive topics (e.g., privacy) and are used on a vulnerable population has raised ethical questions (Howes & Gastmans, 2021).

To gain a better understanding of how various stakeholders view the topic of ETDs in dementia care, several qualitative studies have been conducted, with the majority focusing on end-users of devices: PwDs, their families, professional caregivers, or other healthcare professionals (Berridge & Wetle, 2019; Cooper et al., 2019; Gullslett et al., 2021). Developers of ETDs have received little attention in the literature. Existing qualitative research with developers centers on exploring adoption, acceptability, and usability of ETDs (Freiesleben et al., 2021; Mahoney & Mahoney, 2010; Neubauer et al., 2022). No studies to our knowledge have investigated developers’ perceptions of the ethics related to the design, development, and use of ETDs.

Developers are important stakeholders. Choices made during development have an impact on end-users, and many scholars argue that the development process is not ethically neutral but shapes the morality of technology (Friedman et al., 2002; Verbeek, 2006). The critical role of developers is further bolstered by examples of normative articles offering suggestions to developers to make ETDs more ethically sound (Bennett et al., 2017; Mangini & Wick, 2017; Meiland et al., 2017). The ongoing ethical discussion surrounding ETDs is incomplete without the unique viewpoints and expertise of developers, whose contributions could offer new insight into the ethics of ETDs in dementia care. Therefore, the aim of this study was to gain a deeper understanding of ETD developers perceptions related to the ethics of their work. To achieve this, our research question was: *How do developers of ETDs perceive ethical issues surrounding the design, development, and use of ETDs within dementia care?*

## Methods

### Study Design

We used a qualitative study design based on a constructivist account of grounded theory (Charmaz, 2014; Singh & Estefan, 2018). This design is well suited to exploring under-researched areas, as research data are generated abductively via the interaction between the participants, researchers, and their environment (Charmaz, 2008). We followed the COREQ guidelines for reporting qualitative research (Tong et al., 2007).

### Participant Selection and Recruitment

Product development describes a set of intertwined design and development activities (e.g., market research, software design, user interface and user experience research, etc.) generally aimed at improving upon an existing product or creating a new product (Schlattmann & Seibel, 2021). This process occurs within commercial and university settings by individuals or interdisciplinary teams. We use ‘developer’ as a general term referring to persons involved in any aspect of design and development. To reflect the diversity found within development, participants were eligible for inclusion if they: (1) have been involved in any stage of the design and development of an ETD as defined in the introduction; (2) within a university or commercial context; (3) that reached the minimum stage of a working prototype; (4) and were able to be interviewed in English.

Informed by convenience sampling, three active strategies were used to purposively recruit from this niche population (Ellard-Gray et al., 2015; Negrin et al., 2022). The first involved reaching out by email to contact persons at commercial companies and university consortiums based on a list developed from a previous study of commercial ETD company websites (Howes et al., 2022), a search of EU and USA funded research projects, and a general internet search. The second strategy involved sending direct emails to academic researchers who had published articles on ETD prototype development, and messages via LinkedIn to commercial developers. Lastly, snowballing was used, where participants were asked to forward the study brochure to colleagues who might be interested. Study materials (study brochure, General Data Protection Regulation Rights packet, informed consent document, socio-demographic questionnaire) were given to potential participants, who were encouraged to read them and ask any questions before submitting a signed informed consent to JH.

### Data Collection

Data collection occurred through 15 individual, semi-structured interviews conducted over Microsoft Teams by JH, a 29 year-old male Ph.D. student with a background in Bioethics and Theology. Data collection started in November 2021 and concluded in November 2022. A semi-structured interview approach was chosen because it facilitates in-depth exploration of a particular concept or experience, and therefore is well suited to gaining insight into this under-researched topic. An initial interview guide (supplementary material 1) was developed based on previously completed research (Howes et al., 2022; Howes & Gastmans, 2021; Vandemeulebroucke et al., 2022) and a pilot interview with an industrial engineer working in product development. The interview guide was abductively refined throughout data collection, integrating insights from completed interviews and early data analysis.

Participants were interviewed via video call; however, two participants preferred audio-only interviews. Overall, no major technical problems occurred, and participants located themselves in quiet, private spaces without major distractions. The average interview length was 62 min, with a range of 42–78 min. With the consent of participants, the interviews were recorded and subsequently transcribed verbatim. Per the study protocol, all video and audio recordings were destroyed at the conclusion of the study, though interview transcripts were retained. No field notes were made. Access to primary data was restricted to the main researcher JH and was stored on a KU Leuven server.

### Data Analysis

**Fig. 1:**
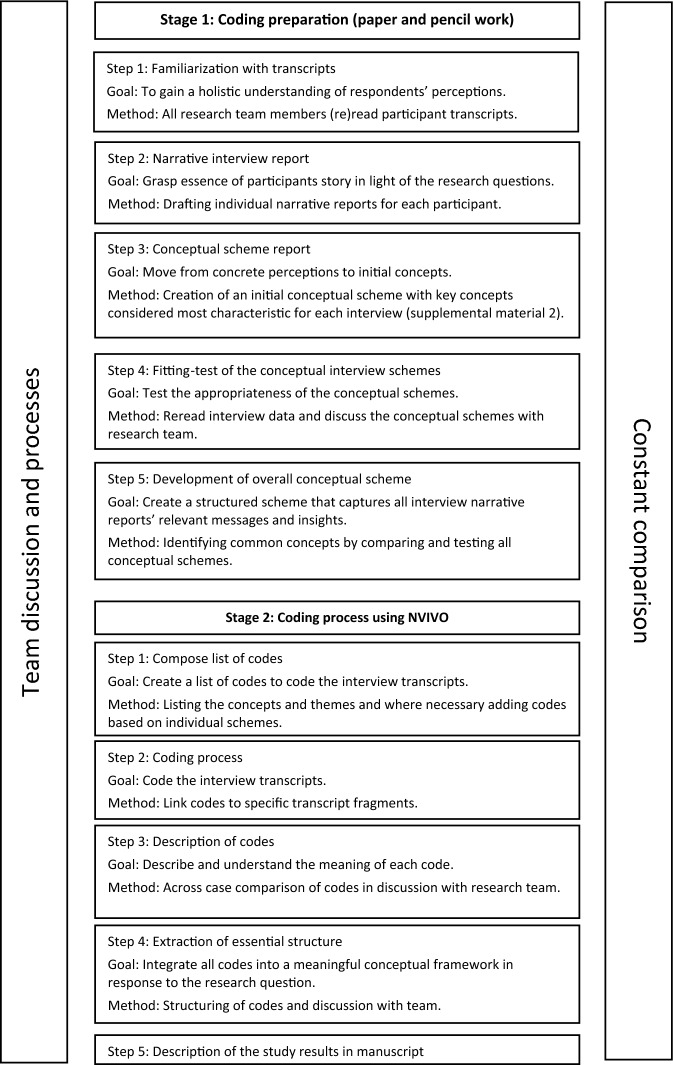
Overview of the Qualitative Analysis Guide of Leuven data analysis process. Each step entails team discussions and continuous comparison between cases and previously completed steps. This figure is inspired by Vandemeulebroucke et al. (2020)

Data analysis was conducted according to the Qualitative Analysis Guide of Leuven (QUAGOL) (Dierckx de Casterlé et al., 2012; Dierckx de Casterlé et al., 2021), as it can provide structure to the two-step data analysis approach of constructivist grounded theory, which consists of an initial broad coding, followed by a close, focused coding (Rieger, 2019; Vandemeulebroucke et al., 2020). The QUAGOL, an abductive data analysis method, threads between a purely inductive or purely deductive approach. It consists of two stages with five steps each, and is characterized by an iterative forward–backward movement between all stages and steps (Dierckx de Casterlé et al., 2021). This constant movement facilitates the use of prior knowledge to engage with the data and generate theory that can be tested and refined through further data collection and analysis. The first stage involves a comprehensive pre-coding preparation process using only pencil and paper–no qualitative software is used. The second stage involves the actual coding process, using qualitative software (Dierckx de Casterlé et al., 2021). The QUAGOL requires that researchers engage in in-depth and sustained interaction with data, ensuring that generated theory stays close to participants’ experiences. The analysis process is always conducted within an ongoing dialogue among the research team. An overview of the stages and steps of QUAGOL are found in Fig. 1.

The data analysis was conducted by an interdisciplinary team composed of the interviewer and three bioethicists with extensive experience and expertise in qualitative ground theory research. The pre-coding steps were independently completed by JH, YD, TV, and CG and were discussed in regular team meetings. Differences of view on concepts or themes were discussed until a consensus was reached. To enhance reflexivity, at key points in analysis one researcher would be intentionally excluded from a meeting, being brought in later to independently present their findings to evaluate and fit check the completed work. The second stage of QUAGOL was completed by JH using the qualitative analysis software NVIVO (QSR International; released 2020), JH's work was continually evaluated and discussed with YD, TV, and CG.

### Ethics

The study methodology and all study materials were approved on September 2nd, 2021, by the Social and Societal Ethics committee of KU Leuven (File: G-2021-3903).

## Results

### Participant Demographics

We contacted 137 persons, 53 being academic researchers and 84 being commercial developers. Out of 26 replies, 9 declined and 17 agreed to participant, however, 2 dropped out before their interview, leaving a total of 15 included participants, 13 male and 2 female (Table 1). Most participants were from North American and Western European countries. The participants’ ages ranged from 29 to 71, with most being between 45 and 55 years old. Participants self-reported their roles in development as being involved: in all aspects of the process; project management or management of the overall design process; and direct involvement in specific aspects broadly consisting of software, hardware, manufacturability design, or end-user engagement. A diverse and varied professional background was present and while largely divided into technical and business, many participants reported having experience in both areas. At the time of the interview, most participants had 11–20 years of working experience.

**Table 1:** Participant demographics

Characteristics	Participants (N = 15)
*Sex*	
Male	13
Female	2
*Age*	
26–35	3
36–45	2
46–55	8
56–65	1
65 +	1
*Country*	
United States	3
Denmark	3
Austria	2
Germany	2
Canada	1
Belgium	1
United Kingdom	1
Turkey	1
Singapore	1
Professional background*	
Technical (e.g., engineering, computer science)	8
Business (e.g., finance, marketing, change management)	8
Other (e.g., sociology, education)	4
*Years of working experience*	
2–10	4
11–20	8
21–30	1
30 +	2
*Professional setting*	
Commercial company	9
University	6
*Entry into ETD development*	
Hired into development	6
Dialogue with stakeholders	6
Personal experience with dementia	3
*Part of development*	
All aspects	5
Management	5
Specific aspects (e.g., software, hardware)	3
End-user engagement	2

*Participants may be represented in more than one category

### Results Overview

**Fig. 2:**
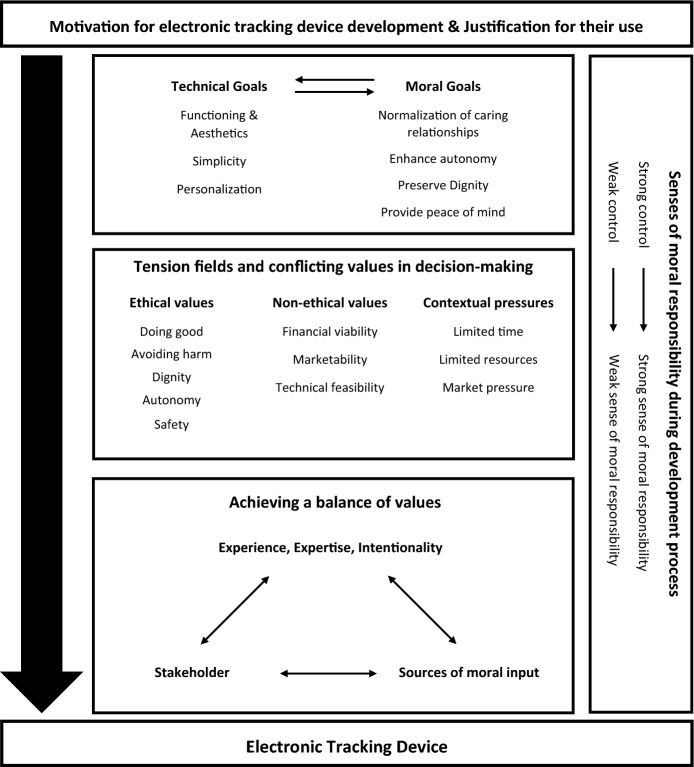
Conceptual overview of results. This figure illustrates the interrelations between result themes, focusing on participants ethical perceptions of the development process. It depicts a progression from initial motivation, justification, and goal identification to confronting ethical tensions and achieving balance, with sense of moral responsibility shaping the entire process

In discussing the ethical issues surrounding their work, participants shared stories about their personal and professional journey from initial awareness of wandering-related challenges to applying their expertise to improve PwD and caregivers’ quality of life and the challenges they confronted along the way. Common points of perception related to the ethics of ETD development were observed from these stories. These points centered around: (1) the motivation for developing ETDs, (2) the technical and moral goals, (3) the conflicting values encountered during development, (4) the process of achieving a balance of values, and (5) perceptions related to moral responsibility. A conceptual overview of the results is available in Fig. 2, and additional supporting quotes are available in Table 2.

**Table 2:** Illustrative quotes

**(1) What motivates pursuit of ETD development and how do developers justify ETD use?**	
Motivated to intervene for the good	P2-C*: [F]inally were able after a few hours to find him at a gas station not too far from the house. But it was just, you know, calling the cops, everyone getting scared. Trying to figure out what was happening. […] it was a shock to me, so as you know, there is so much great technology that we have out there and I have worked for so many years in technology […] and I said, ‘Well how can I use my knowledge of having worked in that space and in product development and create something that’s differentiated, easy to use, and helps families, gives them a little more of a peace of mind, knowing that if someone with dementia were to wander, they would know about it right away.
ETDs are justified in certain context	**P3-C:** These people [PwDs] are vulnerable in the way that children are vulnerable. Nobody in their right mind would let a 5-year-old child wander around the street so why would you do it with somebody with dementia? And it [ETDs] has become, yeah, acceptable in people's eyes. Thank goodness actually. Because I think if we didn’t have these sorts of devices […] [m]aybe today anybody with dementia would just be locked up. And that would be that.
ETDs are unjustified in certain context	**P1-C:** Its [our ETDs use context] quite explicitly not institutional. Because if people go into memory care, those facilities are typically on lockdown. So its rare that anybody really wanders. They might wander down the hallway but they are not going to get farther than that.
**(2) What technical and moral goals are sought?**	
Evaluation of current ETD offerings	**P11-U:** Well, it largely had to do with in the first instance recognizing the inadequacy of the devices that existed. Then recognizing that in certain circumstances, controlling wandering behavior was desirable. And thus trying to design devices that both did something useful but didn't interfere in any way with dignity with the practices of carers.
Technical goal: Simplicity	**P2-C**: So it goes back to creating something that is K.I.S.S. We all know, ‘Keeping it simple stupid.’[…] Keeping it easy for them to use and to understand.
Technical goal: Personalization	**P12**-U: [The geo-fence] should be personalized. I mean some geofence is needed to be adapted to the person, to the patient herself or himself and there [I] came into the personalization idea.
Moral goal: Increase PWD’s safety and enhance autonomy	**P1-C:** So if a PWD still has certain things that they are kind of allowed to do on their own, go for a walk around the block, this is a typical kind of consideration I think for dementia patients, that they ugh, you want them to have as much autonomy as they can, safely.
Moral goal: Giving caregivers peace of mind	**P8-U:** Actually it's a stress relieving for —a reliever for the nurses or for the caregivers. Knowing, OK if I don't hear anything, everything is OK.
Moral goal: Enhance dignity	**P12-U:** The 1st generation of systems. Actually didn't take into account the dignity [of PwDs]. That's where the problems started to occur. […] Dignity is something very, very personal. That's why […] systems must be personal.
Moral goal: Restore normalcy to PWD-caregiver relationship	**P3-C:** With our device we hope that people get some extra time with the family. And even if it just gives them a few months to, really just come to terms with things, it is something.
**(3) Conflicting values in decision making**	
*(3a) Conflicting ethical values*	
Doing good and avoiding harm	**P3-C:** [W]e know that people with dementia can just throw the device in a bin. Because they don’t understand why they got it. They just decide—and we knew that [not storing data on the device] was important. That if somebody got a hold of it and decided that they were going to somehow take it apart or going to try and hack into it. We didn’t want anybody compromised[.]
Autonomy and Safety	**P13- C:** The right to continue to live your life and the right to have autonomy is […] the highest for the individual. But there's a balance between the safety, privacy and autonomy that that just happens with the space
Promote dignity and avoid stigmatization	**P13-C:** this stigma around, somebody gets dementia and then they are dead or they are less of a person is just false. So how can you reverse that and figure out how to empower them instead of […] pushing them down?
*(3b) Conflicting non-ethical values*	
Balance decisions with financial solvency	**P2-C:** And the caveat that we do behind all of this is, we take the feedback, we listen to it, we try and look at what we can and can’t do. And three, is we implement what we can in appropriate timelines. Taking into account that I am trying to run a business[.]
Profit as non-absolute value	**P1-C:** [O]k, so we’ve got this wonderful tracking technology, what could we do? People would say, well, you know you can provide it for spouses who are trying to track their adulterous spouse, you could turn it into a dog collar for dogs who run away. […] But [its] just not part of our mission.
Lack of market visibility	**P3-C:** One of the really big problems in this industry is, people don’t know about it. People don’t know that these devices exist.
**(4) Achieving a balance**	
*(4a) Stakeholder inclusion in development*	
Stakeholder inclusion is important	**P1-C:** I think that this kind of work is almost always a dialogue between developers and users. […] And I think that process of feedback is absolutely crucial.
Co-Creation	**P7-U:** [W]e do a lot of Co-creation. It's very explorative in the beginning of a concept. We do a lot of those things and they (partners) did not really mention that in the proposal, they talked about surveys and interviews, and that's OK, but with Co-creation it's still a little bit different and a bit more interactive. With mockups and prototypes and so on. You already try something before there really is a real product already.
Traditional marketing methods	**P15-C:** So we did kind like try to [go into] the forums and click into the Facebook groups for the tracker space. We actually look into both like the dementia trackers and also pet trackers. So there's a larger pet community that also requires something similar. So then we started asking people, hey this tracker you used and what happened and so on[.]
Need to interpret feedback	**P9-C:**. Yeah, and the end users, the primary end users, so the elderly people and especially the care persons. They had ideas that we realized later on that that is not even asked for, it was too personal from Uh, from those persons involved, yeah. But the markets said no we don't need this, yeah it has to be easier. It has to be very lean. Everything has to be lean. Yeah, yeah, not too many possibilities to change processes. Yeah, it should be a.. how I say? Not as flexible as we thought it should be, yeah.
*(4b) Sources of moral input*	
Company mission	**P5-C:** I think all people in [company name] feel that they have a moral and ethical responsibility to have a safe device for the market. So everybody is having that mindset. So I don’t see any persons in the company just doing their work. *deep sigh* So people are extremely concerned about what happens at the customer [level]…There is the thinking, which should be in a medical device company, always think of the customer […] I am not coming in and saying, ok now I should ensure that laser engraving works and then I leave again. No. In that, we always have the discussion of, if I do this what is then the user experience?
Domain experts	**P3-C**: Yes. We absolutely as a company say that we are not the experts in this because we are selling the product and we talk to people about the product and we talk to people doing research into dementia, but because—thank god— we are not experiencing it ourselves, we are not the expert on that side of things. That is why we will always go and ask other people about it.
**(5) Perceptions of moral responsibility**	
(*5a) Strong feelings of responsibility*	
Professional obligations	**P5-C:** But it was especially on discussing, how to say, the robustness for the product and design for manufacturability. And ensuring a high level of… yeah of course when you look at production you look at low scrap rates, lowest cost as possible, blah blah blah in all parameters. And there we had quite some work to do. […] I came up with all the irritating things that we had to do this and that, and we need to do these tests for water proof-ness and drop tests.
Responsibility to bring intentionality to work	**P7-U:** [T]hat it is important in all the ethics steps before you start to study. To think well about it, how you would engage with them [end-users], how they would consent to what you plan to do. That's really important, not only going to a commission, a medical ethical commission with the board that gives it a yes.
Responsibility to anticipate and prevent introducing problems	**P13-C:** I think every technology will be misused. Every technology will be hacked. You have a responsibility to think about those things and know that as the ground truth when you're building it. So that you can mitigate as much as you can. If you build an entire thing and then think about security at the last minute. Then you completely misled, completely lost everyone, complete. You know it's just a failure completely.
(5b) Weak feelings of responsibility	
Realistic power to enact change	**P11-U:** It’s hard to think of anyone who would argue I have no ethical responsibilities. I mean, of course it comes down to how limited are those responsibilities? I mean, I have to say the cynic in me says that we often exaggerate the power of academics to make any kind of difference. […] That's my cynical answer. My other answer says something like you have to recognize the limits of your responsibilities on a practical level. There's a limit to what you can achieve.
Reliance on expert decisions	**P9-C:** On our department for civil law. Uhm, they said it's no need, actually, to [include PWD in long term testing]. And if there is no need, we shouldn't put the pressure on those people. So there are other ways to come to the results we needed. So we shouldn't include them.
No control over use of devices	**P14-C:** Yeah, I don't have any control of how the system is used in the end, that's. That's not my. That's not part of my responsibility. They have to go to a judge and sign up for fixing –fixating the person. So if the judge says yes, then I'm OK with that.
(5c) At the boundaries of responsibility: Misuse of ETDs	
Misuse as inevitable	**P13-C:** This idea around you know… Technology misuse and all that, I think every technology will be misused. Every technology will be hacked.
ETDs perceived as neutral tool	**P6-C:** As a manufacturer, we have to step back and say, OK, well, we're responsible for providing a system to help prevent this patient from leaving this residence. But […] we're there as a tool. We're not there to replace the vigilance of the staff because they still have to pay attention to everything that's going around them.
Challenge of balancing prevention with user experience	**P1-C:** [I]t is tricky, because so you don’t want to stigmatize people, by having them feel like they are constantly being watched over, so we don’t make the app very visible […] [b]ecause we don’t want them [PWD] […] constantly being reminded that their [being tracked], or wondering, ‘what is this? Why am I getting pinged like this?’ On the other hand it’s also not totally invisible, so there is a little icon […] and if you tap it you can see some information about it. So you know, [s]omebody who does not […] want to be tracked, could do that. And so that’s a kind of balance that we have tried to strike.
Provide professional insight to customers	**P14-C:** When a nursing home comes to me and asked me […] how it's best to get the tech to the dementia patient. I advise them, for example, to involve the family. So come to make it as positive as possible, that maybe [the] daughter or son comes to the mother or father and gives the tag [ETD] as a gift, for example, or that the tag [ETD] is in the same shape as a watch so that there is no stigmatization wearing something on the wrist. […] That they get used to it and that the product is as positive as possible, but this is always up to the nursing home. How they solve it, but I advise them to that.

*C: Company-based participant

#U: University-based participant

### (1) What Motivates the Pursuit of ETD Development and How Do Developers Justify ETD Use?

Participants expressed a strong feeling of moral responsibility to use their expertise to create ETDs that would improve the lives of PwDs and caregivers. Whether introduced to dementia-related wandering through direct experience caring for PwDs, being approached by dementia care stakeholders, or joining active development efforts, most participants highlighted a moment where they became aware of immense burdens related to wandering, saw the need for a better solution, and recognized that they possessed the requisite expertise to possibly build a better technical solution.

In the motivation to pursue ETD development, there was a recognition of the potential benefit of location technologies for dementia care. This potential formed the main justification developers held for the use of ETDs: that they stand to maximize benefits and minimize burdens for PwDs and caregivers, relative to alternative care options. A common example of this favorable balancing was that ETD use in the home was a preferable alternative to “locking up” PwDs through institutionalization or physical confinement. However, some participants identified moments where this balancing made ETD use unjustified. For some who developed ETDs for at home use, the relative security offered by care institutions staffed with professional caregivers meant that ETDs were unnecessary within institutional care settings. One participant warned that ETD use could be unjustified based on the impact to caregivers, negatively altering their care practices.

“At its worst. It [ETDs] just turns them [PwDs] into objects of control. Oh, Fred^1^ has wandered. Let's go and get him back, you know? This isn't, what does Fred want or need […] It has a consequence in terms of the way in which you, as a carer, behave […] it becomes more of a kind of management problem. Not [a] human problem.” (P11 — University)

### (2) What Technical and Moral Goals are Sought?

The core moral goal sought in ETD development was the increase of PwDs’ autonomy, bolstering of their safety, and the lessening of caregivers’ burdens. Participants’ evaluations of the current ETD offerings on the market were largely pessimistic, deeming them too outdated, stigmatizing, or disconnected from the realities of caregiving. In response, participants discussed the technical and moral goals they pursued to bring innovation to the market and create better devices. Technical goals were related to aesthetics and functioning, whereas moral goals revolved around the impacts that participants hoped to achieve with their devices.

Most technical goals centered on improving the basic device functioning, such as improving battery life, location accuracy, refining software features, and overall simplifying device operation. Simplicity and personalization were frequently emphasized as key technical goals. Often, these specific technical goals were tied to corresponding moral goals, enhancing functionality while also advancing desired moral outcomes. For example, developing improved alarm sensitivity that reduces false alarms to enable a more personal and dignified experience for PwDs. The specific technical and moral goals pursued for PwDs and their caregivers collectively supported broader moral aims concerning the relationship between PwDs and caregivers. The most common broad moral aim was the restoration of normalcy to the lives of PwDs and their caregivers. By providing a simple and customizable device to meet the needs of wandering, participants hoped to grant caregivers peace-of-mind and allow PwDs to remain living their chosen lifestyle for longer, thereby improving their overall quality of life.

“I would hope that it would allow people to focus on the relationship rather than focus on the disease. That is what happens so much with caregiving, is that you get wrapped up in all of the minutia of the disease, and it's like I gotta get food on the table, and I gotta get the, you know, the dog walked, and I got to buy diapers, and all of these things, and wouldn’t it be nice if you were able to set a little bit of that aside and say, ‘hey! I am free to go into the kitchen and prepare something’ and I don’t have to worry that, you know she is out gardening and that is great, she loves to garden, and if she wanders off I’ll get an alert. And just improve the quality of the experience of both parties.” (P1 — Company)

Although participants generally shared the same moral goals, their technical approaches to achieving these often varied significantly, reflecting differing visions for integrating ETDs into care. On a higher level, some participants wanted their device to be unobtrusive, blending into the background of life, noticeable only during emergencies. Others wanted more conspicuous devices that make their presence known to promote continuous awareness of a PwD’s status. Differences in form factors stemmed from what participants thought PwDs would find valuable, useful, and dignified. A notable example concerned the decision to include a locking mechanism on wristbands. One participant completely rejected the use of locking wristbands, whereas another discussed their new keyless locking mechanism as a way to provide a more dignified user experience.

“We don't sell those wristbands where you can't open them anymore. You can, well you can put those wristbands on our products as well, but we don't sell it. The reason is we have seen in a lot of projects that that leads to very aggressive situation or […] can lead to an aggressive situation. […] I put it on myself for 24 hours. It's horrible. If you have something that you're not able to take off. Yeah, it drives you crazy or it drives me crazy. And they [PwDs] just live in the same moment and they have the same situation and they are probably driven crazy as well. So that's why we don't sell this.” (P9 — Company)

“At the end of the day, you need something that's going to be on the person 24/7 that has an opportunity to lock on if you want. […] So you either put it around their neck which leads to unsafe things, like when they're sleeping, or you stick it on their ankle, which leads to them feeling like a prisoner. Or you stick it on the wrist, which is your only other option. […] [S]o we invented a lock that requires two hands to take it on and off. […] So It doesn't use a technology, doesn't use a key. It looks like just a regular watch clasp, but it's hard enough for the individual that they can't do it on their own, and they really need someone else to do it for them. […] [Locking it] needed to happen without technology or a key […] for their [PwDs] own dignity.” (P13 — Company)

### (3) Tension Fields and Conflicting Values in Decision-Making.

The diversity in approaches to achieving moral goals in ETD development emerged from navigating a landscape filled with ethical tensions and conflicting values. While striking a balance amongst all values was important, two primary areas of conflict emerged related to ethical and non-ethical values.

#### (3a) Conflicting Ethical Values

Participants were sensitive to the presence of tension between ethical values and strove to strike an appropriate balance. The primary tensions were between doing good and avoiding harm on the one hand, and autonomy, dignity, and safety on the other.

The primary tension participants spoke about was between doing good and avoiding harm. They recognized that while ETD functionalities offer benefits, these same functionalities could also introduce new risks and vulnerabilities. Further, participants shared the opinion that the risk of harm could be reduced but not eliminated. Participants discussed potential risks related to device misuse, device operation disrupting care practices, or causing stigmatization; however, the most common risk was related to data privacy. Nearly all participants touched on the balance between data privacy/security and the benefit of tracking features. The challenge was finding the right technical balance that would allow the intended good to be achieved while reducing concomitant risks as much as possible.

Participants also encountered tension among key ethical values, primarily consisting of autonomy, dignity, and safety. For the most part, determining their general approach to balancing these values was relatively straightforward, if not self-evident, with many participants having linked their chosen technical innovations to a better balancing of these values. For example, promoting autonomy by allowing a PwD to call and indicate that they are safe when an alarm sounds.

“But the first functionality, I mean the call. Yeah it was. It was, uh. … it occurred from the moment I talked to my advisor. OK, these people, they wander, and OK, I need to make them pay for their freedom. They want freedom. So how do I do that? They need to acknowledge that they are in good health.” (Participant 12 – University)

Rather, the main challenge lay in making detailed decisions regarding which values to emphasize. As seen in one participant’s description of a tiered geo-fence alert system that categorized a PwD’s location into three general zones based on various parameters: within the geo-fence, outside the geo-fence but within an accepted threshold, and outside the geo-fence and in danger. The location of a PwD would only be shared if they are determined to be in a danger zone, with the goal of bolstering their autonomy, reducing false alarms, and promoting privacy.

“OK, you have some kind of pre warning and they have a certain time that the user [PwD] is allowed to stay in or certain distance so that we, […] by simply taking the change in position […] say OK, this is now a dangerous situation and this is not [a dangerous situation]. […] How large can this buffer zone be? How long is somebody outside of his usual environment to be assumed to be in a dangerous situation? We did not address this and maybe by purpose, because this would then raise medical questions that we could not answer.” (P9 — University)

#### (3b) Conflicting Non-Ethical Values

One area of significant challenge was the balancing of values related to business. These values encompass aspects necessary for the successful management of a company and the success of a particular product within a market. Participants working in commercial settings felt immense pressure to ensure all decisions were balanced with the need to ensure financial profit. This pressure led participants to value what they thought would drive customers to purchase their product, potentially leading to other important values being overshadowed, as one participant described in relation to exploring sustainable materials.

“[T]he customers. They do not really care about it [sustainable materials], at this stage. So they wouldn't. They wouldn't pay for it. They wouldn't see it as a surplus or a benefit for the product. […] Regarding environmentally friendliness, there is some room for improvement. I'm very aware of it, but yeah, it's always a matter of is it possible to sell it?” (P14 — Company)

While the need to ensure financial profit was highly weighted, it was not an absolute value. There were moments where participants prioritized other values, specifically over profit maximization. For example, by refusing to sell their product in lucrative adjacent markets like pet tracking or in offering multiple form factors to provide options for PwDs’ preferences.

“And usually it's from a business point, commercial perspective, it's horrible to have so many different product variations, but we do that to make sure that the patient is wanting to wear the tech.” (P14 — Company)

At the same time, participants were sensitive to the pricing of their ETDs, keenly aware that their customers often had limited means. Thus, the accessibility of their product had to be incorporated into the balancing of values.

“Into the cost wise that's the other thing we look at, right? […] And unfortunately when we go around we actually see a lot of dementia, elderly, families actually they are not very well off, frankly speaking. Right, so a lot of them are really living on the edge.” (P15 — Company)

Connected to the pressures of financial profit and accessibility were difficulties in gaining market awareness. Participants discussed how it was difficult to reach their target end users, with some commenting that people, in general, do not know ETDs exist for dementia care. In response, participants described the need to balance decisions with an eye to what would draw in and entice end-users, impacting both ETD features and marketing approaches. For example, one participant felt compelled to use marketing language they would have preferred to avoid.

“[W]hen we start [to] look at what people search for when they should search for a product like ours. Nobody searches for dementia product for finding people. They will search for trackers and GPS’ and all that. […] [W]e shouldn’t be compared with that. But for example, sales wise, we couldn’t not say GPS, so we also say GPS with our product because nobody will find us if we didn’t add GPS to the search term.” (P4 — Company).

Participants had to make decisions regarding the preceding values under the additional pressure of working with limited resources. For university-based developers, this mainly consisted of working within the allotted time and budget. Whereas for commercial-based participants, there was the additional pressure to bring a viable product to market as quickly as possible. In both cases, a prioritization of features to develop was required.

### (4) Achieving a Balance of Values

Participants wanted to create ethically sound ETDs, and in their pursuit of this goal, they relied on several sources to help guide decision-making amidst conflicting values. Foremost among these were the voices of stakeholders, complemented by two sources of moral input, organizational mission and domain experts.

#### (4a) Stakeholder Inclusion in Development

All participants emphasized the importance of including stakeholders in development, deeming it one of the most critical aspects of their work. Stakeholders were seen as possessing unique knowledge and expertise relevant for ETD design, and participants sought their input to better comprehend needs, desired features, useability considerations, and to determine the right balance of features. For many participants, the inclusion of stakeholders also held a moral dimension.

“One is the ethical reason. And again, it comes back to conceptualizing stakeholders a lot more than simply people who are suffering from dementia. I think they have a right to be included in these processes, but equally important is that they have knowledge and experience which is relevant to the design of not just tracking devices, but all sorts of devices that might improve the lives of older people.” (P11 — University)

Participants shared the view that stakeholder engagement was ideally an iterative and ongoing dialogical process. While various engagement methods were discussed, two general groups based on development context emerged. Participants from a university setting or with university partners mainly described methods of co-creation or participatory design, whereas participants from a commercial setting described more traditional business methods, such as market research or surveys. Additionally, participants from commercial settings often described receiving feedback through informal interactions with stakeholders.

“[I]t's more informal so we do get from our support line we get feedback. Yeah, and so that's more the formal process, I'd say, but eventually it's during talks with our partners and during talks with their customers. [...] Yeah, it's more on a personal basis. It's not structured.” (P9 — Company)

In discussing the role of stakeholder inclusion in development, many participants brought up the need to interpret stakeholder feedback in light of their own professional experience, particularly when it came to what was considered marketable. As end-users may not fully understand or appreciate the technical aspects of an ETD.

“So you go in with all these assumptions […] and some of it's obviously going to be wrong. Regardless [of] how much research you did, because you listen to all your customers and they said these things and you try to build what they said, but they don't actually want what they said. So then you gotta go reiterate it again.” (P13 — Company)

One participant discussed the difficulty related to recruiting PwDs, as dementia often advances too quickly for new prototypes to keep up. Additionally, three participants discussed the challenge of identifying the makeup of relevant stakeholders and the complexities involved in balancing all stakeholders’ wants and opinions.

#### (4b) Sources of Moral Input

Participants spoke about two important sources of moral input that helped guide their decision-making: organizational mission and domain experts.

Organizational mission, articulated as a focus or general orientation, was discussed as an important input source that guided decision-making. This mission, for company founders or co-founders, was often tied to their personal sense of moral identity. Other participants articulated it as a collective culture reinforced through daily interactions and dialogues with colleagues. To a lesser extent, participants in an academic context discussed mission in relation to professional integrity.

Participants in both commercial and academic development settings discussed seeking domain experts to help navigate value conflicts. Those in a commercial setting discussed voluntarily seeking expert insight from a variety of professionals (e.g., law, cybersecurity, occupational therapy). Additionally, many participated or sought advice and feedback from informal peer groups. University-based participants likewise sought out expert input but had an obligation to work with their institution’s legal departments and ethics committees to vet their work. For some participants, this mandatory consultation was procedural, akin to a compliance check. Whereas others describe a dialogical process with back-and-forth debate preceding mutual understanding and agreement.

### (5) Perceptions of Moral Responsibility

Participants’ perception of their moral responsibility was often linked to their perception of control. Stronger feelings of responsibility were related to professional obligations, bringing intentionality to design decisions, and the proactive anticipation and mitigation of potential harms. They felt weaker feelings of moral responsibility primarily concerning the use of their products, when they doubted their ability to influence organizational decisions, or when they deferred to relevant authorities or expert figures. The conflicted borders between feelings of responsibility were highlighted when participants spoke about ETD misuse.

#### (5a) Strong Sense of Moral Responsibility

Participants from a range of backgrounds strongly expressed the importance of their professional obligations. Acting in accordance with the best practices of their respective fields helped ensure stakeholders were treated with respect throughout the development process and that the final devices operated in a safe and correct manner. These varied obligations centered on good process and included topics such as design practices, quality control, and informed consent for end-user engagement. In discussing the importance of these obligations, a few participants underscored that PwDs' lives were at stake in the results of their work.

“[S]o you tell everybody you're responsible for lives. If the product doesn't work. Someone might die. It's harsh. Saying it like this, but it's obvious. Yeah, people are relying on the products. And you have to keep that in mind.” (P9 — Company)

Many participants felt that a level of intentionality should be brought to their work. This involved reflecting on the intended use of an ETD, its purpose for being created, how it and the data it generates will be used, and how to best interact with end-users during development.

“[I]f you never build a product where you start to ask customers what they really need, if you never think about what the intended use is, then it is certain that you will have problems later on. Especially with privacy. […] So you need to be aware from the start of what you really need. […] Because it is also important that we don’t just have a lot of devices in the world. But that we have devices that are built for, again, with a purpose.” (P4 — Company).

This intentionality was important to participants, forming a crucial aspect of their responsibility to proactively anticipate and mitigate potential risks introduced by ETDs. This included assessing how design elements might adversely affect end-user experience, such as flashing charge indicators disturbing PwD’s sleep. The main effort, however, was directed toward recognizing ways ETD designs could expose end-users to external risks. Identification of potential misuse encompassed discussion on accidental misuse by end-users, like PwDs inadvertently discarding their device, as well as deliberate malicious misuse, including data security breaches or the harmful use of tracking functions. Participants felt their responsibility extended to thoroughly considering the implications of their choices and implementing them in a manner that mitigates the risk of harm to end-users. As seen in a participant’s discussion of the possibility of allowing third-party volunteers to participate in the use of an ETD, concerns are raised about how this potentially exposes PwDs to being taken advantage of by these volunteers.

“A person with dementia might be easily tricked or I'm not quite sure about the word but you might be tricked or you might be taking advantage of (them). […] So you should not bring the person with dementia to their home, but to like a police station or a home for elderly people or something like this instead.” (P10-University)

#### (5b) Weak Sense of Moral Responsibility

Participants had a weaker sense of moral responsibility in areas they perceived as beyond their control. For some, this was linked to skepticism about their actual ability to influence organizational decisions (i.e., company or university consortium). Others placed their trust in authoritative figures or subject matter experts, such as legal judges or ethics committees, deferring to their decisions. Nearly all participants felt that the use of devices was outside their ability to control and ultimately outside of their responsibility. Once a device is in the hands of users, it is nearly impossible to influence how it will be used, with any possibility to intervene potentially requiring ethically dubious actions on the part of participants, such as the surreptitious surveillance of customers. In articulating this inability to control use, participants described ETDs as neutral tools, emphasizing that the ethical dimension largely lay in their usage, often drawing parallels to kitchen knives.

“[Y]ou are taking people at their word that they are buying the device for the right reasons. And again, it is a bit like selling a knife. If you are selling a kitchen knife to somebody, your assumption is, your hope, whatever you want to call it, is that they are going to use it to chop vegetables and not stab somebody. … We can’t manage every situation.” (P3 — Company)

### (5c) At the Boundaries of Responsibility: Misuse of Electronic Tracking Devices

These boundaries of responsibility came into focus when participants discussed misuse. This boundary was not well defined but characterized by tension between the inevitability of misuse, participants’ responsibility to proactively prevent misuse, and their user experience goals.

Misuse of technology was seen as an inevitable occurrence. While participants believed that the development process should incorporate design choices to minimize misuse, this needed to be balanced with the goals of a device. Participants were hesitant to sacrifice what they saw as core user experiences for the sake of misuse prevention.

“[I]f you ask me, OK. Can it be less likely to be used as a[n] [illicit] privacy tracker? So why not let me make it bigger? But then it defeats the purpose. Right. Or why can't you just make the battery life shorter. So you can't track the person for five days. Then it defeat the purpose.” (Participant 15 – Company)

Communicating proper ETD usage through marketing materials and user guides, as well as providing professional insights to customers, were often discussed as the last avenue for shaping ETD use and preventing misuse. Although ultimately, what end users choose to do with devices are their responsibility.

I'm telling them [customers] the benefits and the negative effects, but … It's their topic, so I'm not taking the responsibility for that. I control and take the responsibility that we don't sell it [locking wrist bands]. But if they are doing this [using locking wrist bands], I tell them OK, from our point of view, this is maybe not the optimal way of handling this. (P9 — Company)

## Discussion

This study provides insight into developers’ ethical perceptions related to the design, development, and use of ETDs in dementia care. Participants felt strong moral obligations related to professionalism and risk management, largely perceiving these duties along a development—use divide. Yet their experience of navigating ethical conflicts suggests this divide to be nuanced. Participants’ ethical reasoning was shaped by a blend of internal factors, such as their professional experiences and personal values, as well as external factors, namely stakeholder interests and difficult business environments. In this mutual shaping of the technical (development) and social (use), ethical, technical, and business values are balanced in conjunction, in a process grounded in concrete experiences rather than abstract principles.

### Challenging the Neutrality Thesis

It was evident that the boundary between development and use formed the demarcation for what participants viewed as being within their purview. They were responsible for decisions made during development, and end-users were responsible for how devices were used. This sharp divide was vividly illustrated through the re-occurring analogy of kitchen knives to portray ETDs as neutral tools. While developers have responsibilities related to professionalism (e.g., quality control, informed consent) and risk management (e.g., anticipate and pre-empt introducing harms), moral responsibility mainly rests on how these tools are used. This view is echoed in recent research on the ethical agency of artificial intelligence developers (Griffin, Green, & Welie, 2023). This concept that technology is neutral, having no built-in tendency to be good or bad, and its ethical impact arising from how it is used, is known in the philosophy of technology literature as the neutrality thesis (Morrow, 2014). In line with this view, the role of ETD developers can be likened to shipbuilders who must construct a resilient, well-functioning vessel, before entrusting end-users with its operation. This perception presents a boundary between the technical (development) and social (use). However, participants’ experiences reveal this boundary to be more nuanced than perceived.

Participants’ strong experience of powerlessness over end-user use and misuse of ETDs exposes a potentially under-acknowledged element of ETD development: the goods sought via ETDs are only attainable via social organization, whether through informal networks of family and friends or formal care organizations (Spilker & Norby, 2019a). An ETD is useless without someone effectively operating it. The technical and social cannot be entirely separated. Indeed, these devices are not created in isolation and then handed over to end-users, rather, an interplay of *mutual shaping* occurs between technology development and social organizations (Oudshoorn & Pinch, 2003). Participants’ experiences show that end-users influence the technical and moral goals developers pursue. This influence is manifested through their wants, feedback, and willingness to purchase ETDs. Conversely, developers promote certain uses of their devices and discourage others based on more than end-user feedback, incorporating a synthesis of their own personal experiences, expertise, and values. This is seen not only in pursuit of certain design elements but also in the dialogue with end-users, aimed at conveying best practices of ETD use.

This dependence on social organization to realize sought for goods, like increased safety, also provides critical insight into the conceptualization of ETDs within dementia care. Device features, such as location tracking, geo-fencing, or fall detection, do not *direct*ly achieve goods (Spilker & Norby, 2019a). Instead, they enhance caregiving practices to enable the achievement of goods. Consequently, it is more appropriate to conceptualize ETDs as *facilitators* of good care, rather than as solutions to wandering. This conceptualization should spur deeper reflection on the integration of ETDs into care environments and under what conditions their use are ethically justifiable (Nordgren, 2018).

### Stakeholder Inclusion as an Emerging Professional Norm

One of the central ways this mutual shaping unfolds is through the inclusion of stakeholders in the development process. Participants from a range of backgrounds in both university and commercial contexts widely agree on the importance of including stakeholders, particularly PwDs, in the development process. This consensus strongly suggests that the principle of stakeholder participation (inclusive of PwDs) in the development of dementia technology, advocated for in the academic literature (Suijkerbuijk et al., 2019), is increasingly experienced as a professional norm. While participants generally describe this inclusion as an iterative dialogue with end-users, no standard method of inclusion was articulated. The diversity of stakeholder inclusion methods may be related to the diversity of participants’ professional backgrounds (Griffin et al., 2023). This diversity may be further compounded by differing healthcare models in participants’ countries, which may enable or hinder recruitment of participants, as our sample included individuals from countries with more socialized healthcare systems and individuals from more privatized healthcare systems.

### It Remains a Business

The participants also faced a challenging business environment that significantly impacted their decision-making processes. Key aspects shaping this environment included the end-users’ lack of awareness about ETDs, limited operational capital, and the ongoing pressure to maintain financial viability. These challenges, as identified in our study, confirm the findings of Freiesleben et al. (2021), where similar factors were discussed as barriers to ETD adoption in focus groups involving ETD developers. Our study goes further, providing initial insight into how developers balance these factors with ethical values. Often, the pressure to ensure financial viability does overrule other values, such as environmental sustainability. This may explain the discrepancy between ETD companies’ online marketing material excluding PwDs as a target audience (Howes et al., 2022; Vermeer et al., 2019), despite developers’ strong emphasis on including them in the development process. The immense pressure commercial participants felt to produce profit may lead to financial viability being viewed as an absolute obligation to be maximalized (Friedman, 2007). However, participants treated it more as a prima facie obligation, which, beyond a certain point, yields to more pressing claims, as illustrated by the examples of upholding dignity or preserving organizational mission over profit motive.

### Integrated Ethics

When integrating these external factors into their ethical reasoning, developers’ own perspectives and experiences played a prominent role in navigating value conflict. Participants’ justification of ETD use stands on the superior benefit-to-burden ratio offered compared to alternative options, and they seek to improve upon this ratio in their own products with a particular sensitivity to ethical tensions related to autonomy, privacy, safety, dignity, and stigmatization. This justification and these ethical perspectives are largely consistent with other stakeholder groups (Cooper et al., 2019; Howes & Gastmans, 2021; Berridge & Wetle, 2019; Gullslett et al., 2021). While some formal ethics approaches, such as university ethics committees and risk assessments, are used, the prevailing sense is that ethical reasoning was integrated into participants' work in ways that did not necessarily use formalized structures. This potentially entails doing ethics without “doing” ethics. Ethical reasoning unfolds from concrete experiences and interactions, not from the level of abstract principles, similar to what Vandemeulebroucke et al. (2020) refer to as “normativity in place.” Ethical decision-making occurs alongside development and business considerations, and this proximity fosters a degree of variability. Ethical values, rather than being considered in isolation—such as evaluating a form factor solely for its capacity to maximize autonomy while ensuring safety—are integrated with additional factors. These include the preceding considerations of financial viability and end-user preferences, as well as technical feasibility and project timelines, all while maintaining a commitment to upholding autonomy and safety. Part of this variability arises from developers’ personal and professional experiences (e.g., what is technically feasible), and part arises from the broader social context (e.g., end-user preferences). This variability could account for diverse technical solutions that purportedly pursue the same ethical values, as exemplified by the antithetical approaches to locking wristbands that pursued the preservation of PwDs’ dignity. This result adds richness and credence to previous research that suggested developers of dementia technology may be implicitly, rather than formally, addressing ethics in their work (Ienca et al., 2018).

The insight gained from exploring developers’ perspectives on the ethics of their work fills a gap in the literature and highlights avenues for future research. The general professional norms that developers share, like stakeholder inclusion or pre-empting risk, should be further confirmed and specified. A closer examination into the role different levels of social organization plays in facilitating or hindering pursuit of ethical values should be undertaken. This closer examination could yield insight into better delineation of responsibility between developer, end-user, and wider health authorities. For instance, a better understanding of how informal care networks (e.g., composed of family and friends) shape and support ETD use may clarify whether and to what extent developers should promote certain conceptions of informal care via their products. Finally, ethical tools should be developed that scaffold, complement, and enhance the dialogical and iterative process of ethical reasoning developers use.

## Strengths and Limitations

This study is the first to focus on the ethical perceptions of ETD developers, bringing a previously underrepresented voice to the wider field. To enhance the trustworthiness of our study, we implemented various strategies (Creswell & Miller, 2000). (1) A comprehensive audit trail was maintained for each research step. (2) Interviews were recorded and transcribed verbatim. (3) A robust and rigorous data analysis method was used, involving prolonged and deep interaction with the research data. (4) Researcher triangulation was used to take advantage of our team size. The study was further strengthened by a thorough recruitment process, as developers are a difficult to reach population for a variety of reasons, and we note that previous studies contained limited numbers of developers (e.g., 2–7 persons) (Freiesleben et al., 2021; Mahoney & Mahoney, 2010; Neubauer et al., 2022).

While our study has several strengths, we also recognize potential limitations. Due to the small sample size, theoretical sampling was not feasible; therefore, only convenience sampling was used, and this introduced potential biases. Our sample may have been biased toward developers who had an interest in ethics and were comfortable discussing their work and its ethical dimensions. Our sample may also underrepresent junior-level employees and those working in business-to-business settings (e.g., contract manufacturing or software development). Additionally, women were underrepresented, although this may be related to general trends within the technology industry (Hill, Corbett, & St Rose, 2010). While one-on-one interviews reduce the risk of socially acceptable answers, the sensitive nature of the subject matter may have influenced participants to provide responses that align with social norms. Future research should focus on exploring potential discrepancies between the ideal and real-world practice of stakeholder inclusion in ETD development. For example, by identifying which social organizations are being engaged or partnered with by commercial developers, and which are not.

## Conclusion

This study provides insight into the way in which ETD developers experienced a strong moral commitment in their work. A key aspect of navigating ethical conflict was stakeholder inclusion, and while this was experienced as a general professional norm, no specific methodology of inclusion was present. Developers tend to perceive their moral responsibility as limited to areas within their control, mainly centering on professional standards and risk management, leading to a distinction between the development and use of ETDs. This distinction is not clear cut, however, and future research should evaluate the role social organizations have on ETD development and use, as well as specify the general norms experienced by developers from differing backgrounds.

### Supplementary Information

Below is the link to the electronic supplementary material.

Supplementary file1 (PDF 214 KB)

Supplementary file2 (PDF 682 KB)

## Footnotes

1 This example is hypothetical and does not pertain to any specific individual.
