# Supplementary material for: The Ethics of Electronic Tracking Devices in Dementia Care: An Interview Study with Developers
Source: Sci Eng Ethics. 2024 May 8;30(3):17. doi: 10.1007/s11948-024-00478-0 (PMC11078786; doi:10.1007/s11948-024-00478-0)
Supplement: Supplementary file 1 — Supplementary file1 (PDF 214 KB) [file 11948_2024_478_MOESM1_ESM.pdf]

Supplemental material 1: Interview guide

Title: The ethics of electronic tracking devices in dementia care: An interview study with developers

Interview Guide

1. Presentation
2. Reference to the information brochure
3. Reference to the informed consent
4. Ask if they have further questions
5. Ask permission to record the interview

Introduction

Electronic tracking devices (ETDs) —also known as locators, monitors, or personal safety alarms— are technological tools that facilitate the monitoring and/or logging of a person with dementias' (PWD) real-time and historical location. A common example being a fob or watch that contains GPS functionality.

ETDs are being used within dementia care to help manage wandering, a behavioral symptom of dementia that involves frequent, repetitive, and temporally and/or spatially disordered movements that are closely associated with eloping from safe areas. Elopement may turn into becoming lost, leading to situations with high risk of serious injury or death. Various ethical questions have been raised regarding ETDs in dementia care and several studies have sought input from key stakeholders (e.g. Older persons, (in)formal caregivers, health professionals, etc.); however, to date little research has focused on individuals involved in the design and development of ETDs. Therefore, developers' perceptions, experiences, and opinions regarding the ethics of ETDs are largely absent from the literature.

That such a prominent stakeholder group has been left out of the literature is concerning, particularly because of the growing recognition that technology design has a major impact on user behavior, as well as on the ethical evaluation of said technology.
Therefore, an accounting of developers' perceptions regarding the ethics relating to the design, development, and use of ETDs is necessary.

What is missing from the literature? Your perspective.

Therefore, the research question of this study is:
How do developers perceive the ethical issues surrounding the design, development, and use of electronic tracking devices in dementia care.

We would like to ask you some questions to gain a better insight on this subject.

GO OVER INFORMED CONSENT.

-Do I have your consent to record this conversation?

Warm up/opening questions

- Why did you decide to participate in this study?
- How did you end up working on electronic tracking devices?

Setting the stage

- Can you recall a previous ETD that you helped develop?
    - How would you explain the overall design and development process to an outsider?
    - What was your role in this process?
- What language do you use to describe your product?

Questions about Design and Development:

In thinking of this past ETD that you worked on…

- How does this device solve problems?
- How did you identify problems in need of solving?
- How did you decide on what problems to focus on?
- How did you decide (or take part in deciding) on features to include in the final product?
- Who is this device for?
- How and why did you decide on the form factor that you did?
- Do you think there was any tradeoffs that had to be made in the design of this device?
- How did you test your device?
- What were the challenges you faced in designing/developing this ETD?
- Do you think that you have ethical responsibilities related to your work?

Questions about USE:

- How do you think the ETD impacts the relationship between persons with dementia and their caregivers?
  - Why should a person with dementia want to use your device?
  - Why should a caregiver want to use your device?

- How do you hope people use your device?
- Could someone misuse your device?
- What happens to your device when an individual no longer needs it?
- What is your intention regarding the device end of life?

- Do you think that designers and developers have ethical responsibilities related to how their device will be used?
  - Do you feel like you had responsibilities related to the use of your device?

Concluding questions

- How do you feel about working on a project like this?
  a. [did you accomplish what you set out to accomplish?]
  b. [If you could go back, what would you change about the design/development process of your companies ETD?]

- If you were put in charge of making sure the next ETD from your company was ethical, how would you accomplish this?

- Anything else you want to talk about or would want others to know?
