# Supplementary material for: The Ethics of Electronic Tracking Devices in Dementia Care: An Interview Study with Developers
Source: Sci Eng Ethics. 2024 May 8;30(3):17. doi: 10.1007/s11948-024-00478-0 (PMC11078786; doi:10.1007/s11948-024-00478-0)
Supplement: Supplementary file 2 — Supplementary file2 (PDF 682 KB) [file 11948_2024_478_MOESM2_ESM.pdf]

Supplemental material 2: Example conceptual scheme.

Title: The ethics of electronic tracking devices in dementia care: An interview study with developers.

Participant X Conceptual Scheme update #6 –April 29th, 2022

Research Question: "How do developers of ETD perceive ethical issues surrounding the design, development and use of ETD within dementia care?

Main points:

- Practice oriented
- Morality is inherent to technology
- Law is distinct from morality
- Personal experience as motivation
  Relational orientation of ETD (should have impact on relation between PWD and caregiver).

1. **Moral Goals (Intended impact Tech has on user experience of world)**
    a. Moral Goal
        i. Return normalcy to PWD/Caregiver relationship. **(Value:** Relationships**)**
        ii. Peace of mind for caregivers **(Value:** Beneficence**)**
        iii. Maximize autonomy for PWD **(Value:** Autonomy**)**
        iv. Reduce stigmatization caused by ETD (**Value** Dignity; **Value:** Autonomy).
        v. Maximize safety for PWD. **(Value:** Safety**; Value: Beneficence)**
        vi. Personalization **(Value: Person centered care)**
    b. Ethical Concepts
        i. General method of ethical Evaluation— Paternal Beneficence (need to balance sometimes conflicting needs of caregivers and PWD).
        ii. Ethics inherent with technology (ETDs fraught with 'tricky gray areas')
        iii. Company has a mission.
        iv. Strong feeling of moral agency (e.g., draw line on what device is used for, even if others would distinguish line elsewhere.)
2. **Technical Goals (Intended technical functionality necessary to achieve moral goal).**
    a. Customizable safe zones (caregiver can tailor make -zones/that PWD can freely move within).
    b. Attractive/stylish form factor (apple watch)
    c. Tap into existing technology features (oxygen sat, vitals, etc.)
    d. Better battery life
3. **Design and Development process (To realize intended technical and moral goals – how to get "there").**
    a. Formal Process
        i. Design
            1. Choice of underlying technology infrastructure (e.g. Apple/Samsung/proprietary) — Based on personal experience with the technology.
                a. Limits/hinders design possibilities
                b. Frees/bolsters design possibilities
            2. Identification of needs
                a. Based on personal experience.
                b. Based on stakeholder feedback.
        ii. Development
            1. Stakeholders (Primarily caregivers)

            a. Dialogical relationship between stakeholders and developers.
            b. Stakeholder involvement essential to development.
            c. Stakeholders contribute to refinement of core technology.
    iii. Use
        1. Intended Use context — Informal Care Setting.
        2. Intended User — Informal Caregivers (family of PWD).
        3. Unintended/misuse of Device.
            a. Unintended Use — N/A
            b. Malicious Miss-Use
                i. Limited responsibility to prevent misuse
                ii. Efforts to prevent misuse needs to be balanced with impact on user experience (i.e., PWD).
- b. Informal Process
    - i. Personal experience driven process
    - ii. Informal 'club' of peers provide discussion and feedback (doctors, developers,etc.)
    - iii. Iterative Internal discussions with team members regarding design decisions (e.g., how to mitigate potential for device misuse).

4. Social-Historical Context wherein ETD development takes place

- a. Personal experience
    - i. Lived experience of caring for PWD (vulnerability of PWD/caregivers)
    - ii. Experience in technology development
    - iii. Feel a responsibility to intervene for good
    - iv. Creative desire to help vulnerable people
- b. Societal Context
    - i. Organization of Healthcare
        - 1. Non-socialized health system (more private/for-profit)
        - 2. Trust in formal institutions to manage wandering.
- c. Business Context
    - i. Company has a mission
    - ii. Extremely difficult business environment
    - iii. Reliance on 3rd party companies
    - iv. Business orientation: Technology development came first, then marketing second.
- d. Technology Context
    - i. All technology is limited
        - 1. Deliberate limitation introduced by creator of existent technology.
        - 2. Inherent limits of a technology (e.g. technically impossible to do X). .
    - ii. Dissatisfaction with current ETD market offerings
    - iii. Reliance on already existing technology
- e. Legal Context
    - i. Currently no legal framework for ETD use.
